# Comparison of CKD-EPI Cystatin C and Creatinine Glomerular Filtration Rate Estimation Equations in Asian Indians

**DOI:** 10.1155/2014/746497

**Published:** 2014-04-27

**Authors:** Boon Wee Teo, Charumathi Sabanayagam, Jiemin Liao, Qi Chun Toh, Sharon Saw, Tien Yin Wong, Sunil Sethi

**Affiliations:** ^1^Department of Medicine, Yong Loo Lin School of Medicine, National University of Singapore and National University Health System, 1E Kent Ridge Road, Level 10 NUHS Tower Block, Singapore 119228; ^2^Singapore Eye Research Institute, c/o Singapore National Eye Center, 11 Third Hospital Avenue, Singapore 168751; ^3^Department of Ophthalmology, Yong Loo Lin School of Medicine, National University of Singapore and National University Health System, 1E Kent Ridge Road, NUHS Tower Block, Singapore 119228; ^4^Office of Clinical Sciences, Duke-NUS Graduate Medical School, National University of Singapore, 8 College Road, Singapore 169857; ^5^Department of Laboratory Medicine, National University Hospital and National University Health System, 5 Lower Kent Ridge Road, Singapore 119074; ^6^Ophthalmology and Visual Sciences Academic Clinical Program, Duke-NUS Graduate Medical School, National University of Singapore, 8 College Road, Singapore 169857; ^7^Department of Pathology, Yong Loo Lin School of Medicine, National University of Singapore and National University Health System, 1E Kent Ridge Road, Level 10 NUHS Tower Block, Singapore 119228

## Abstract

*Background*. Chronic kidney disease (CKD) is identified in the general population using estimated glomerular filtration rates (eGFR) calculated from a serum creatinine-based equation, the chronic kidney disease-epidemiology collaboration (CKD-EPI) equation. Using serum cystatin C in combination may improve eGFR accuracy. We evaluated the new CKD-EPI equations incorporating cystatin C in a population of Asian Indians in classifying CKD across body mass index, diabetes, and hypertension status.* Methods*. We retrieved standardized serum creatinine and serum cystatin C data from a cohort of 2877 Asian Indians aged 40–80 years from the Singapore Indian Eye Study and calculated eGFR (in mL/min/1.73 m^2^) with the new CKD-EPI equations and serum creatinine only equation.* Results*. The creatinine only equation mean eGFR (88 ± 17) was similar to using spline Log cystatin C (88 ± 22). The lowest mean eGFR (81 ± 21) was obtained with the spline Log cystatin C—age, sex, and weight equation. The creatinine only equation had the fewest participants (7.1%) with eGFR <60 and spline Log cystatin C—age, sex, and weight equation had the most (16.1%).* Conclusions*. Using serum cystatin C resulted in widely varying eGFR which significantly affected the classification of chronic kidney disease.

## 1. Introduction

Chronic kidney disease is identified in the general population using estimated glomerular filtration rates (eGFR) calculated from formulae [1]. Serum creatinine-based equations, such as the Modification of Diet in Renal Disease study equation [2] and the Chronic Kidney Disease-Epidemiology Collaboration (CKD-EPI) equation [3], are commonly used for clinical care and research. However, serum creatinine levels are affected by non-GFR factors such as muscle mass (body composition), diet, and medications [4]. It has been shown that, using another filtration marker, such as serum cystatin C, in combination with serum creatinine in prediction equations, the accuracy of GFR estimation is improved [5–7]. GFR estimation using serum cystatin C may be affected differently by obesity (body fat distribution). The CKD-EPI group recently published the combination of serum creatinine and cystatin C equations and other serum cystatin C-based equations which incorporated other variables such as weight or diabetes [8]. In this study we assess GFR estimation using these equations in an ethnic Asian-Indian population and examine their performance in relation to body mass index, diabetes, and hypertension.

## 2. Methods

### 2.1. Study Population

The present study utilized data from the Singapore Indian Eye Study (SINDI), a population-based cross-sectional study of 3,400 Indians aged 40–80 years, conducted from 2007 to 2009 with detailed methodology reported elsewhere [9]. In brief, from a computer-generated random list of 11,616 Indian names provided by the Ministry of Home Affairs, 6,350 adults were selected by an age-stratified random sampling method. Of the 4,497 eligible participants, 3,400 participated in the study with a response rate of 75.6%. For this analysis, we included those with data on serum creatinine and serum cystatin C measurements (*n* = 2877).

### 2.2. Laboratory Assays

We retrieved stored serum and urine samples for assays performed in a central clinical laboratory. The assays were traceable to standardized reference materials using manufacturer provided calibrators where applicable. Creatinine concentrations were determined by the Jaffe method on the Beckman DxC800 analyzer, with manufacturer provided calibrators traceable to SRM 967. The concentration of serum cystatin C was measured using particle-enhanced immunoturbidimetric assay calibrated with materials traceable to ERM-DA471/IFCC, and urine albumin was assayed with a PEG-enhanced immunoturbidimetric method on the Siemens ADVIA 2400 platform (http://www.siemens.com).

### 2.3. Measurement of Covariates

Age was defined as the age at the time of examination and was categorized into 2 groups: 40–65 and >65 years. Body mass index (BMI) was calculated as weight in kilograms divided by the square of height in meters (kg/m^2^). BMI was categorized into <20, 20–25, 25–30 (overweight), and ≥30 kg/m^2^ (obese). Diabetes mellitus was defined as a casual plasma glucose ≥200 mg/dL (11.1 mmol/L) or self-reported physician-diagnosed diabetes or use of glucose-lowering medication. Hypertension was defined as systolic BP ≥ 140 mm Hg or diastolic BP ≥ 90 mm Hg or self-reported previously diagnosed hypertension.

### 2.4. Statistics

We estimated GFR (eGFR) using the CKD-EPI equations, where Scr is serum creatinine and Scys is serum cystatin C, and all the study participants were treated as “white” [3, 8]. 

*(1) Serum Creatinine 2009 Equation (Age, Sex, and Race) [3]. *Consider 141 × min⁡(Scr/*κ*,1)^*α*^ × max⁡(Scr/*κ*,1)^−1.209^ × 0.993^Age^  [×1.018  if  female]  [×1.159  if  black], where *κ* is 0.7 for females and 0.9 for males, *α* is −0.329 for females and −0.411 for males, min is the minimum of Scr/*κ* or 1, and max is the maximum of Scr/*κ* or 1.

*(2) Serum Cystatin C 2012 Equation (Age and Sex) [8]. *Consider 133 × min⁡(Scys/0.8,1)^−0.499^ × max⁡(Scys/0.8,1)^−1.328^ × 0.996^Age^  [×0.932  if  female], where min indicates the minimum of Scr/*κ* or 1 and max indicates the maximum of Scys/*κ* or 1.

*(3) Serum Creatinine in Combination with Cystatin C 2012 Equation (Age, Sex, and Race) [8]. *Consider 135 × min⁡(Scr/*κ*,1)^*α*^ × max⁡(Scr/*κ*,1)^−0.601^ × min⁡(Scys/0.8,1)^−0.375^ × max⁡(Scys/0.8,1)^−0.711^ × 0.995^Age^  [×0.969  if  female]  [×1.08  if  black], where *κ* is 0.7 for females and 0.9 for males, *α* is −0.248 for females and −0.207 for males, min indicates the minimum of Scr/*κ* or 1, and max indicates the maximum of Scr/*κ* or 1.

*(4) Average of the Serum Creatinine and the Serum Cystatin C Equations [8].* GFR were also estimated with the other cystatin C equations in the supplementary material which were in the format of spline Log equations and additional adjusters of age, sex, race, history of diabetes, and weight, where min indicates minimum of standardized Scys/0.8 or 1 and max indicates maximum of standardized Scys/0.8 or 1. The units of cystatin C, age, and weight are in mg/L, years, and kg, respectively [8]. 

*(5) Spline Log Cystatin C Equation [8]. *Consider 109 × min⁡  (Scys/0.8,1)^−0.683^ × max⁡  (Scys/0.8,1)^−1.367^.

*(6) Spline Log Cystatin C, Age, Sex, and Race Equation [8]. *Consider 132 × min⁡(Scys/0.8,1)^−0.491^ × max⁡(Scys/0.8,1)^−1.329^ × 0.996^Age^  [×0.932  if  female]  [×0.992  if  black].

*(7) Spline Log Cystatin C, Age, Sex, and Diabetes Equation [8]. *Consider 126 × min⁡(Scys/0.8,1)^−0.362^ × max⁡(Scys/0.8,1)^−1.318^ × 0.997^Age^  [×0.934  if  female]  [×1.068  if  diabetes].

*(8) Spline Log Cystatin C, Age, Sex, and Weight Equation [8]. *Consider 132 × min⁡(Scys/0.8,1)^−0.567^ × max⁡(Scys/0.8,1)^−1.329^ × 0.996^Age^  [×0.949  if  female] × 1.002^Weight−80^.

First, we compared the characteristics of the study participants by categories of BMI using chi-square test or ANOVA as appropriate for the variable. Second, we estimated mean eGFR across BMI categories by all estimating equations. Tests for linear trend across BMI categories were performed using BMI categories as an ordinal variable in linear regression model. Third, we compared mean eGFR by diabetes and hypertension status for all equations and assessed the significance in difference by Student's* t*-test. Fourth, we estimated the mean difference in eGFR of the various equations compared to the CKD-EPI creatinine 2009 equation. Fifth, we calculated the proportion of participants falling into each eGFR category (>90, 60–90, 30–60, 15–30, and <15 mL/min/1.73 m^2^) for all equations. Finally, using the CKD-EPI creatinine 2009 equation as the reference, we compared the concordance of CKD categories defined by all other equations and also compared the direction of eGFR change among the discordant categories. We did not consider the Modification of Diet in Renal Disease (MDRD) study equation because the study population is based on a general population and includes both CKD and healthy people without kidney disease. Moreover, estimates of GFR using the MDRD study equation can only be reported as >60 mL/min/1.73 m^2^ for those with better function. It is an important function of this study to look at the variation of estimated GFR as a continuous variable throughout the range of estimation. Significance was taken at the 5% level. All statistical analyses were performed using STATA version 12.0 (Texas, USA).

## 3. Results

56.7% of the participants were either overweight (39.6%) or obese (17.1%) (Table 1). Obese participants were more likely to be younger, female, shorter, with lower levels of serum creatinine, with higher levels of serum cystatin C, with systolic blood pressure (SBP), and with higher prevalence of diabetes and hypertension. Those with BMI <20 kg/m^2^ (175, 6.1%) were more likely to be older, with lower levels of SBP, with diastolic blood pressure (DBP), and with lower prevalence of diabetes and hypertension.

The creatinine 2009 equation mean eGFR was similar to using spline Log cystatin C and both gave the highest mean eGFR of 88 mL/min/1.73 m^2^ with SD of 17 for eGFR creatinine and 22 for spline Log cystatin C. However, in the obese category (BMI > 30 kg/m^2^), the spline Log cystatin C mean eGFR (83 ± 23 mL/min/1.73 m^2^) was significantly lower than creatinine 2009 mean eGFR (89 ± 19 mL/min/1.73 m^2^) (*P* < 0.001). The lowest mean eGFR (81 ± 21 mL/min/1.73 m^2^) was obtained with the spline Log cystatin C—age, sex, and weight equation. Of all the BMI categories, mean eGFR was higher among subjects with BMI in the 20–25 kg/m^2^ category and lower among obese subjects by all equations except creatinine 2009 (*P* = 0.2) and spline Log cystatin C—age, sex, and weight equation (*P* = 0.8). Compared to BMI in the 20–25 kg/m^2^ category, mean eGFR was also lower in the BMI < 20 kg/m^2^ category. Generally, the inclusion of serum cystatin C resulted in eGFR that were lower than using creatinine 2009 except for the spline Log cystatin C equation.

Mean eGFR was lower in diabetic (except by spline Log cystatin C diabetes) and hypertensive subjects by all equations (Table 2(b)). The lowest mean eGFR was consistently obtained by the spline Log cystatin C—age, sex, and weight equation regardless of diabetes or hypertension status. The highest mean eGFR in nondiabetic patients was obtained with the creatinine 2009 equation (90 ± 16 mL/min/1.73 m^2^), and the lowest mean eGFR (82 ± 19 mL/min/1.73 m^2^) resulted from the spline Log cystatin C—age, sex, and weight equation (*P* < 0.001). The highest mean eGFR in diabetic patients (86 ± 25 mL/min/1.73 m^2^) was obtained using the spline Log cystatin C equation versus the lowest mean eGFR of using the spline Log cystatin C—age, sex, and weight equation (79 ± 23 mL/min/1.73 m^2^, *P* < 0.001). Nonhypertensive participants had higher mean eGFR (ranging from 87 ± 19 to 93 ± 21 mL/min/1.73 m^2^), whereas hypertensive patients had the lowest mean eGFR (77 ± 21 to 84 ± 23 mL/min/1.73 m^2^) (*P* < 0.001 by all equations).

The creatinine 2009 equation had the smallest proportion of participants (7.1%) with eGFR <60 mL/min/1.73 m^2^ and spline Log cystatin C—age, sex, and weight equation had the largest proportion (16.1%) (Table 3). The equations incorporating serum creatinine for GFR estimation had the lowest proportion of participants with eGFR <60 mL/min/1.73 m^2^ (creatinine 2009 = 7.1%, creatinine-cystatin C = 9.2%, and average of creatinine and cystatin C = 8.8%). The cystatin C 2012 equation had more than double the proportion of patients (14.6%) with eGFR <60 mL/min/1.73 m^2^ compared to the creatinine 2009 equation (7%).

When we compared the concordance of CKD stages classified by various cystatin C equations versus CKD-EPI creatinine 2009 equation (Table 4(a)), the concordance was the highest with the average of creatinine 2009 and cystatin C 2012 (78.1%), followed by creatinine-cystatin C 2012—age, sex, and race (77.3%), and was the lowest with the spline Log cystatin C—age, sex, and weight (59.3%). In general, the concordance was high at lower eGFR levels by all equations. Table 4(b) shows the direction of movement of eGFR categories comparing cystatin C equations to CKD-EPI creatinine 2009 equation among those who were different. Movement to a higher category ranged from 5.9% with the average of creatinine 2009 and cystatin C 2012 to 14.8% with the spline Log cystatin C. Movement to a lower category ranged from 16% (average of creatinine 2009 and cystatin C 2012) to 31.5% (spline Log cystatin C—age, sex, and weight). Under the >90 category, 13.2% to 22.8% were originally classified as >90 moved to a lower category by the cystatin C equations. Under the 60–90 category, 5% to 12.5% participants were originally classified to the 60–90 mL/min/1.73 m^2^ category and 0.9% to 2.1% of those in the 30–60 mL/min/1.73 m^2^ category had eGFR changes that resulted in reclassification into a higher GFR category.

**Figure 1:**
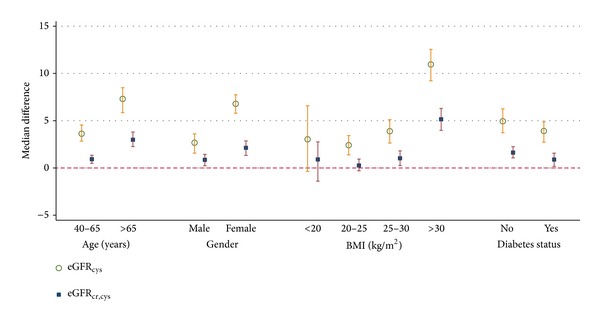
Performance of estimating equations by age, gender, BMI, and diabetes status. Referent equation: serum creatinine 2009 equation (age, sex, and race) [3].

Compared to creatinine 2009 equation, the median difference in eGFR was larger when using the “cystatin C 2012 equation” than the “creatinine in combination with cystatin C 2012 equation” (Figure 1). The differences were the greatest in the elderly (age >65 years), women, obese (BMI >30), and nondiabetic participants.

## 4. Discussion

This is the largest study of a population-based cohort of Asian Indians comparing GFR estimation using a variety of equations incorporating serum creatinine alone, serum cystatin C alone, or in various combinations with other variables (age, sex, diabetes, and weight). In general, GFR estimation using serum cystatin C resulted in lower estimates (except using the spline Log cystatin C equation). Including cystatin C as a predictor resulted in lower estimated GFR in all participants except with the spline Log equation form (which did not consider the other predictors—age, sex, and race). The addition of age and sex as predictors into the spline Log equations lowered mean eGFR. “Diabetes” status increased mean eGFR, whereas “weight” as a predictor had a variable effect on mean eGFR.

Depending on the variables used, GFR estimations vary widely. Obese patients may have relatively reduced muscle mass resulting in lower serum creatinine levels and consequently a higher eGFR. However, we know that obese patients are also more likely to have diabetes and hypertension, conditions which are associated with kidney disease and dysfunction [10]. Incident hypertension and diabetes are also associated with elevated cystatin C levels [10, 11]. Therefore, using serum cystatin C in prediction equations may result in eGFR that could be more reflective of “true” GFR in obese patients. This highlights that different equation predictors (non-GFR determinants of marker concentrations) may substantively influence the resultant eGFR. Weight and obesity are non-GFR determinants and their influence on GFR marker levels may be due to ethnicity-related differences in body composition (percentage body fat and lean muscle mass) [12–16]. Serum cystatin C concentrations may be affected by body composition (fat mass) as the largest differences in GFR estimates occur in the obese (BMI >30 kg/m^2^), women, and the participants aged >65 years. Women in general have more body fat than men relative to muscle mass (influences serum creatinine concentrations). Similarly, older participants have reduced muscle mass and a relative higher proportion of body fat. Lower serum creatinine concentrations result in high estimated GFR; yet the higher serum cystatin C concentrations in women and older participants result in lower estimated GFR using the cystatin C-based equations, thereby accentuating the differences in estimates of GFR.

Consequent to the widely differing eGFR, the identification and staging of CKD are significantly affected. Clinical practice guidelines suggest that persistence of eGFR <60 mL/min/1.73 m^2^ with a duration of more than 3 months might be considered CKD; the different cystatin C prediction equations resulted in different prevalence of CKD detected (8.8% to 16.1%), which varies with the prevalence detected by the creatinine only equation (7%) [1]. Cystatin C >1 mg/L is associated with an increased incidence of adverse outcomes despite having creatinine-based eGFR >60 mL/min/1.73 m^2^ [17], and this may be related to incomplete assessment of risk due to an inaccurate eGFR [18]. Therefore, the addition of cystatin C as a predictor of GFR (which generally reduced mean eGFR in this study) may improve the identification of CKD. This has implications for the identification and treatment of CKD in the individual patient and also for the planning and allocation of healthcare resources for CKD management at the level of public health administration. Therefore, it is important that longitudinal studies are performed to assess the performance of these GFR estimation equations by linking clinical outcomes (end-stage renal disease and deaths), especially in ethnically diverse populations in Asia [13, 19–23].

The strengths of our study include a large population-based single-ethnicity cohort with systematically collected demographic data, clinical history, serum, and urine samples. The study is limited by the absence of a reference standard GFR measurement and therefore cannot determine the “true” accuracy of these equations.

In summary we showed that using serum cystatin C as a predictor of GFR in estimation equations resulted in widely varying eGFR which significantly affected the identification and classification of chronic kidney disease. Further research linking these equations to longitudinal clinical outcomes in other Asian populations is required.

## Figures and Tables

**Table 1:** Demographics and characteristics.

Parameter*	(mean ± SD)	Body mass index (kg/m^2^)				*P* value
	All patients (*n* = 2877)	<20 (*n* = 175)	20–25 (*n* = 1071)	25–30 (*n* = 1138)	≥30 (*n* = 493)	
Age (years)	57.3 ± 9.7	60.3	57.9	56.6	56.4	<0.001
Male (*n*, %)	1501, 52.2	57.7	59.9	52.5	32.9	<0.001
Height (cm)	162 ± 9	163	164	162	160	<0.001
Weight (kg)	69.2 ± 13.6	48.9	61.5	72.1	86.2	<0.001
Body mass index (kg/m^2^)	26.2 ± 4.7	18.4	22.9	27.2	33.7	—
Serum creatinine (mg/dL)	0.87 ± 0.29	0.86	0.88	0.87	0.81	<0.001
Serum cystatin C (mg/L)	0.98 ± 0.27	0.98	0.97	0.97	1.03	<0.001
Urine albumin to creatinine ratio (mg/g)	41 ± 139	34	45	39	42	0.6
Systolic blood pressure (mm Hg)	135 ± 19.8	132	134	136	137	<0.001
Diastolic blood pressure (mm Hg)	78 ± 10.3	75	78	79	77	<0.001
Hypertension (*n*, %)	1597, 55.5	45.1	50.6	57.4	65.5	<0.001
Diabetes (*n*, %)	948, 33.0	21.1	31.2	34.0	38.5	<0.001

*All parameters reported as mean ± SD, or frequency, %. Conversion factor for unit: serum creatinine in mg/dL to *𝜇*mol/L, ×88.4.

**Table:** (a)

Equation	Mean estimated GFR* (mL/min/1.73 m^2^)					
	All patients	Body mass index (kg/m^2^)				*P* for trend
		<20	20–25	25–30	>30	
Creatinine 2009—age, sex, and race	88 ± 17	87 ± 18	88 ± 17	88 ± 17	89 ± 19	0.2
Cystatin C 2012—age and sex	83 ± 21	83 ± 23	85 ± 21	83 ± 20	78 ± 21	<0.001
Creatinine-cystatin C 2012 (age, sex, and race)	86 ± 19	85 ± 20	87 ± 19	86 ± 18	84 ± 20	0.02
Average of creatinine 2009 and cystatin C 2012	85 ± 17	85 ± 19	86 ± 17	85 ± 17	84 ± 23	0.03
Spline Log cystatin C	88 ± 22	89 ± 2	90 ± 22	88 ± 22	83 ± 23	<0.001
Spline Log cystatin C—age, sex, and race	82 ± 21	82 ± 23	84 ± 20	83 ± 20	77 ± 21	<0.001
Spline Log cystatin C—age, sex, and diabetes	85 ± 20	84 ± 22	86 ± 20	85 ± 20	80 ± 21	<0.001
Spline Log cystatin C—age, sex, and weight	81 ± 21	78 ± 22	82 ± 20	82 ± 20	79 ± 22	0.8

*Estimated GFR reported as mean ± SD.

**Table:** (b)

Equation	Mean estimated GFR* (mL/min/1.73 m^2^)					
	Nondiabetic	Diabetic	*P* value	Nonhypertensive	Hypertensive	*P* value
Creatinine 2009 (age, sex, and race)	90 ± 16	85 ± 20	<0.001	92 ± 15	84 ± 18	<0.001
Cystatin C 2012 (age and sex)	84 ± 19	80 ± 23	<0.001	88 ± 19	78 ± 21	<0.001
Creatinine-cystatin C 2012 (age, sex, and race)	87 ± 17	83 ± 22	<0.001	91 ± 16	82 ± 19	<0.001
Average of creatinine 2009 and cystatin C 2012	87 ± 16	82 ± 20	<0.001	90 ± 15	81 ± 18	<0.001
Spline Log cystatin C	89 ± 21	86 ± 25	0.009	93 ± 21	84 ± 23	<0.001
Spline Log cystatin C—age, sex, and race	83 ± 19	80 ± 23	<0.001	88 ± 19	78 ± 21	<0.001
Spline Log cystatin C—age, sex, and diabetes	84 ± 19	86 ± 24	0.01	89 ± 18	81 ± 21	<0.001
Spline Log cystatin C—age, sex, and weight	82 ± 19	79 ± 23	<0.001	87 ± 19	77 ± 21	<0.001

*Estimated GFR reported as mean ± SD.

**Table 3:** Distribution of study population by mean estimated GFR categories.

Equation	Distribution* *n*, %					
	Estimated GFR categories (mL/min/1.73 m^2^)					eGFR <60
	>90	60–90	30–60	15–30	≤15	
Creatinine 2009 (age, sex, and race)	1495, 52.0	1177, 40.9	191, 6.6	11, 0.4	3, 0.1	205, 7.1
Cystatin C 2012 (age and sex)	1162, 40.4	1295, 45.0	395, 13.7	22, 0.8	3, 0.1	420, 14.6
Creatinine-cystatin C 2012 (age, sex, and race)	1271, 44.2	1340, 46.6	248, 8.6	15, 0.5	3, 0.1	276, 9.2
Average of creatinine 2009 and cystatin C 2012	1253, 43.6	1371, 47.7	238, 8.3	12, 0.4	3, 0.1	253, 8.8
Spline Log cystatin C	1384, 48.1	1184, 41.2	289, 10.1	18, 0.6	2, 0.1	309, 10.8
Spline Log cystatin C—age, sex, and race	1125, 39.1	1317, 45.8	410, 14.3	22, 0.8	3, 0.1	435, 15.2
Spline Log cystatin C—age, sex, and diabetes	1249, 43.4	1271, 44.2	338, 11.8	17, 0.6	2, 0.1	357, 12.5
Spline Log cystatin C—age, sex, and weight	1072, 37.3	1341, 46.6	438, 15.2	23, 0.8	3, 0.1	464, 16.1

*Distribution reported as frequency count, *n*; percentage, %.

**Table:** (a)

Equation	Estimated GFR categories by CKD-EPI creatinine 2009 equation (mL/min/1.73 m^2^)										
	>90		60–90		30–60		15–30		<15		Total
	Same *n* (%)	Different *n* (%)	Same *n* (%)	Different *n* (%)	Same *n* (%)	Different *n* (%)	Same *n* (%)	Different *n* (%)	Same *n* (%)	Different *n* (%)	Same *n* (%)
Cystatin C 2012 (age and sex)	895 (31.1)	600 (20.9)	706 (24.5)	471 (16.4)	141 (4.9)	50 (1.7)	8 (0.3)	3 (0.1)	3 (0.1)	0 (0.0)	1753 (60.9)
Creatinine-cystatin C 2012 (age, sex, and race)	1114 (38.7)	381 (13.2)	936 (32.5)	241 (8.4)	160 (5.6)	31 (1.1)	10 (0.3)	1 (0.03)	3 (0.1)	0 (0.0)	2223 (77.3)
Average of creatinine 2009 and cystatin C 2012	1110 (38.6)	385 (13.4)	960 (33.4)	217 (7.5)	163 (5.7)	28 (1.0)	10 (0.3)	1 (0.03)	3 (0.1)	0 (0.0)	2246 (78.1)
Spline Log cystatin C	1016 (35.3)	479 (16.6)	681 (23.7)	496 (17.2)	124 (4.3)	67 (2.3)	8 (0.3)	3 (0.1)	2 (0.1)	1 (0.03)	1831 (63.6)
Spline Log cystatin C—age, sex, and race	871 (30.3)	624 (21.7)	710 (24.7)	467 (16.2)	145 (5.0)	46 (1.6)	8 (0.3)	3 (0.1)	3 (0.1)	0 (0.0)	1737 (60.4)
Spline Log cystatin C—age, sex, and diabetes	936 (32.5)	559 (19.4)	702 (24.4)	475 (16.5)	134 (4.7)	57 (2.0)	6 (0.2)	5 (0.2)	2 (0.1)	1 (0.03)	1780 (61.9)
Spline Log cystatin C—age, sex, and weight	840 (29.2)	655 (22.8)	707 (24.6)	470 (16.3)	147 (5.1)	44 (1.5)	10 (0.3)	1 (0.03)	3 (0.1)	0 (0.0)	1707 (59.3)

**Table:** (b)

Equation	Estimated GFR categories by CKD-EPI creatinine 2009 equation (mL/min/1.73 m^2^)											
	>90		60–90		30–60		15–30		<15		Total	
(*N* = number of patients classified differently from CKD-EPI creatinine 2009 equation)	Up *n* (%)	Down *n* (%)	Up *n* (%)	Down *n* (%)	Up *n* (%)	Down *n* (%)	Up *n* (%)	Down *n* (%)	Up *n* (%)	Down *n* (%)	Up *n* (%)	Down *n* (%)
Cystatin C 2012 (age and sex) (*N* = 1124, 39%)	0 (0.0)	600 (20.9)	264 (9.2)	207 (7.2)	39 (1.4)	11 (0.38)	3 (0.1)	0 (0.0)	0 (0.0)	0 (0.0)	306 (10.6)	818 (28.4)
Creatinine-cystatin C 2012 (age, sex, and race) (*N* = 654, 22.7%)	0 (0.0)	381 (13.2)	157 (5.5)	84 (2.9)	26 (0.9)	5 (0.17)	1 (0.03)	0 (0.0)	0 (0.0)	0 (0.0)	184 (6.4)	470 (16.3)
Average of creatinine 2009 and cystatin C 2012 (*N* = 631, 21.9%)	0 (0.0)	385 (13.4)	143 (5.0)	74 (2.6)	26 (0.9)	2 (0.07)	1 (0.03)	0 (0.0)	0 (0.0)	0 (0.0)	170 (5.9)	461 (16.0)
Spline Log cystatin C (*N* = 1046, 36.4%)	0 (0.0)	479 (16.6)	361 (12.5)	135 (4.7)	60 (2.1)	7 (0.24)	3 (0.1)	0 (0.0)	1 (0.03)	0 (0.0)	425 (14.8)	621 (21.6)
Spline Log cystatin C—age, sex, and race (*N* = 1140, 39.6%)	0 (0.0)	624 (21.7)	251 (8.7)	216 (7.5)	35 (1.2)	11 (0.38)	3 (0.1)	0 (0.0)	0 (0.0)	0 (0.0)	289 (10.0)	851 (29.6)
Spline Log cystatin C—age, sex, and diabetes (*N* = 1097, 38.1%)	0 (0.0)	559 (19.4)	309 (10.7)	166 (5.8)	50 (1.7)	7 (0.24)	5 (0.17)	0 (0.0)	1 (0.03)	0 (0.0)	365 (12.7)	732 (25.4)
Spline Log cystatin C—age, sex, and weight (*N* = 1170, 40.7%)	0 (0.0)	655 (22.8)	229 (8.0)	241 (8.4)	34 (1.2)	10 (0.3)	1 (0.03)	0 (0.0)	0 (0.0)	0 (0.0)	264 (9.2)	906 (31.5)
